# A systematic review of the biological effects of resveratrol on venous thromboembolism

**DOI:** 10.22038/ajp.2025.26263

**Published:** 2026

**Authors:** Hasan Momeni, Fatemeh Shirvani-Farsani, Iraj Baratpour, Saeid Heidari-Soureshjani, Catherine MT Sherwin

**Affiliations:** 1 *Department of Oral and Maxillofacial Surgery. School of Dentistry, Isfahan (Khorasgan) Branch, Islamic Azad University, Isfahan, Iran*; 2 *Oral and Maxillofacial Resident at Faculty of Dentistry, Islamic Azad University Isfahan Branch, Isfahan, Iran. *; 3 *Student Research Committee, Shahrekord University of Medical Sciences, Shahrekord, Iran*; 4 *Deputy of Research and Technology, Shahrekord University of Medical Sciences, Shahrekord, Iran. *; 5 *Professor, Department of Pharmacology & Toxicology (Adjunct), Wright State University Boonshoft School of Medicine, Dayton, Ohio, USA*

**Keywords:** Resveratrol, Venous thromboembolism, Deep vein thrombosis, Pulmonary embolism, Thrombosis, Medicinal herbs

## Abstract

**Objective:**

Venous thromboembolism (VTE) has high morbidity in major surgery, serious injury, or during periods of inflammation and infection. VTE has serious complications, resulting in death. This review aims to evaluate the efficacy and mechanisms of resveratrol (RSV) in preventing and treating deep vein thrombosis (DVT) and pulmonary embolism (PE).

**Material and methods:**

Various databases like MEDLINE/PubMed, Embase, Scopus, Cochrane Library, and Web of Science were comprehensively searched to find relevant studies published before January 2024. After defining the inclusion and exclusion criteria, selecting studies related to the purpose of the study, data were extracted, and study characteristics, methods, and biological mechanisms were recorded and reviewed.

**Results:**

RSV potentially prevents and attenuates VTE through antioxidant, anti-inflammatory, and anticoagulant mechanisms. It inhibited endothelial and platelet reactive oxygen species (ROS) production, enhanced endogenous antioxidants, and downregulated nuclear factor kappa B (NF-κB) and proinflammatory cytokines. RSV also regulated coagulation and fibrinolysis, inhibited tissue factor (TF) and myeloperoxidase (MPO), and reduced apoptosis. Additionally, RSV reduced adhesion molecule expression, including vascular cell adhesion molecule-1 (VCAM-1), intercellular adhesion molecule 1 (ICAM-1), P-selectin, and von Willebrand Factor (vWF), while promoting vasodilation and endothelial protection through increased nitric oxide (NO) production, SIRT1 activation, and ANGPT2 expression.

**Conclusion:**

*In vivo* and *in vitro* studies have revealed that RSV has promising effects on DVT and PE. However, more well-designed controlled clinical trials with human subjects are needed to examine its application in clinical settings.

## Introduction

Venous thromboembolism (VTE) is when blood clots form inside blood vessels, reducing blood flow (Hobohm et al. 2021). Deep vein thrombosis (DVT) and pulmonary embolism (PE) are the leading causes of thrombosis mortality around the world (Stone et al. 2017). Venous thromboembolism can lead to leg swelling and sometimes post-thrombotic syndrome, called DVT, and sometimes results in PE, which can be life-threatening, and lead to chronic thromboembolic pulmonary hypertension. Moreover, VTE can occur in splanchnic veins and other unusual locations (Behravesh et al. 2017; Hong et al. 2021). In addition to causing considerable costs to the healthcare system, this disease causes work-related disability and untimely death in affected people (Braekkan et al. 2016; Bui et al. 2020). The treatment of VTE involves anticoagulation therapy. Usually, the standard VTE therapy includes warfarin or vitamin K-antagonists (VKAs), with heparin or fractionated heparin bridging. However, oral anticoagulants have been validated in certain cases to be used instead of warfarin, as per recent clinical trials carried out on a large scale (Hong et al. 2021; Stone et al. 2017). Bleeding, osteoporosis, and impaired renal function are most commonly associated with direct oral anticoagulant or VKA complications (Ageno and Donadini 2018; Martinez et al. 2020; Palareti et al. 2020). The increasing use of herbal medicines and phytopharmaceuticals as alternatives to conventional therapies for VTE highlights the growing demand for safer and more effective treatment options. This trend is particularly significant given the complications associated with widely used antithrombotic medications like warfarin and other oral anticoagulants (Mukherjee and Chattopadhyay 2022). Therefore, it is necessary to adopt effective and safe treatment strategies to treat thrombosis. 

Recently, herbal remedies have gained popularity due to their lower side effects compared to chemical drugs, affordability, detoxifying properties against cancer drugs, and promising outcomes in treating various diseases (Amini Chermahini et al. 2020; Ekor 2014; Jivad et al. 2024; Khaledifar et al. 2023). Resveratrol (RSV) is a polyphenol (trans-3,5,4′-trihydroxystilbene) found in nuts, grapes, cassia, and berries (Moshfegh et al. 2016; Wang et al. 2022). It has several beneficial properties, including anti-inflammatory, antioxidant, and anti-tumorigenic effects (Bohara et al. 2022; Ramírez-Garza et al. 2018). Structurally, RSV belongs to the stilbenoid group, which consists of two aromatic rings linked by an ethylene or ethene bridge (Roy et al. 2022).

In comparison to other phytochemicals that have antithrombotic properties, the RSV-rich diet is notable for its potential to interact with major pathways involved in thrombosis, such as modulating the immune system and modulating inflammatory cytokines. Moreover, its favorable safety profile and well-documented efficacy in supporting cardiovascular health and vascular function further underscore its therapeutic potential (Berman et al. 2017; Gligorijević et al. 2021). In various studies, RSV has revealed cardioprotective, hepatoprotective, anticancer, and antiviral effects due to its antioxidant and anti-inflammatory effects (Izzo et al. 2021a; Planinc et al. 2022; Talib et al. 2020; Wang et al. 2022). 

Considering the high morbidity and mortality associated with VTE and lack of a study to comprehensively review the efficacy and mechanisms of RSV in the treatment of VTE, the present systematic review study was designed and implemented. 

## Materials and Methods

### Search strategy

To conduct an electronic search, we conducted a thorough systematic review on January 15, 2024, using high-coverage biomedical databases, such as MEDLINE/PubMed, Embase, Web of Science (WOS), Cochrane Library, and Scopus. The primary and Medical Subject Headings (MeSH) keywords used for the electronic search were: ((“resveratrol” OR “3,4',5-Stilbenetriol” OR “3,4',5-Trihydroxystilbene” OR “3,5,4'-Trihydroxystilbene” OR “SRT 501”) AND (“thrombosis” OR “venous thrombosis” OR “deep vein thrombosis” OR “deep-vein thrombosis” OR “deep-venous thrombosis” OR “phlebothrombosis” OR “embolism” OR “pulmonary embolism” OR “thromboembolism” OR “pulmonary thromboembolism” OR “pulmonarythromboembolism”)). Searches were supplemented by a reference list review of relevant articles and studies from the prior review in this systematic review aim. The search was refined until all publications in the review were identified by our search. To ensure the accuracy of the research, we imported the peer-reviewed publications into EndNote 21.2 (released on October 17, 2023, by Thomson Reuters). 

### Selection criteria

All clinical and preclinical studies investigating the effects of RSV on VTE (including DVT and PE) in humans and animals were included. We selected clinical trials and observational studies that met the PICO (Patient/Population, Intervention, Comparison, and Outcome) criteria: 

Population (P): Patients/animals/cells with DVT and PE, or those in experimental conditions where thrombosis is induced.

Intervention (I): Current treatment for VTE and RSV administration.

Control (C): Patients without thrombosis.

Outcomes (O): The primary mechanism of RSV in influencing thrombosis outcomes.

We excluded studies such as systematic reviews, meta-analyses, case reports, editorials, and reviews, articles did not have full-text availability, studies without original data, abstract-only publications, conference poster publications, unpublished study protocols, studies published in languages other than English and letters to the editors. 

### Screening and full-text assessment

Two researchers independently reviewed the titles and abstracts of the studies using predefined inclusion and exclusion criteria. Publications that appeared to satisfy the inclusion criteria were listed, and their full-text versions were obtained for further evaluation. The same researchers then assessed the full texts to confirm eligibility. Any disagreements were resolved through discussion with a third reviewer.

### Data extraction and narrative synthesis

The researchers reviewed full texts, and the most essential information related to the effect of RSV on DVT and PE was extracted. For each study, we recorded the first author's name, publication year, study setting, study design, models (participants/animals/cells), inducing thrombosis, dosage of RSV, and duration, registered in an Excel form. 

### Reporting guideline

This systematic review is reported based on the Preferred Reporting Items for Systematic Reviews and Meta-Analyses (PRISMA) 2020 guidelines.

## Results

### Search results

The process for searching relevant studies is shown in Figure 1 with the PRISMA 2020 flow diagram. Initially, the electronic search detected 803 titles and abstracts. After removing 94 articles with duplicate titles, four titles and abstracts were excluded for various reasons. Among these, one article was not published in English (Lou et al. 2018a), two were deemed irrelevant to the aim of this study (Huang et al. 2020; Pendurthi et al. 1999), and one was excluded because the full text was not available for review (WU and Chen 2016).

After careful evaluation and screening of articles, 20 studies were finally included, of which 11 were related to DVT (Banu et al. 2022; Fei et al. 2022; Kirimlioglu et al. 2008; Lou et al. 2017; Lou et al. 2018b; Lou et al. 2021; Lu et al. 2019; Shahidi et al. 2020; Xu et al. 2016; Yao et al. 2019; Zhang et al. 2018) and nine were related to PE (Chun et al. 2012; Dutra et al. 2017; Hsia et al. 2021; Huang et al. 2021; Kim et al. 2016; Lin et al. 2019; Liu et al. 2022; McCreary et al. 2022; Yang et al. 2019).

### Description of the included studies

The studies were all preclinical, and only one study had a clinical trial design. Studies generally reported promising results from using RSV on VTE. The characteristics and consequences related to the effect of RSV on DVT are shown in Table 1, and the characteristics of the studies conducted regarding the mechanisms of RSV in reducing PE complications are shown in Table 2.

**Figure:**
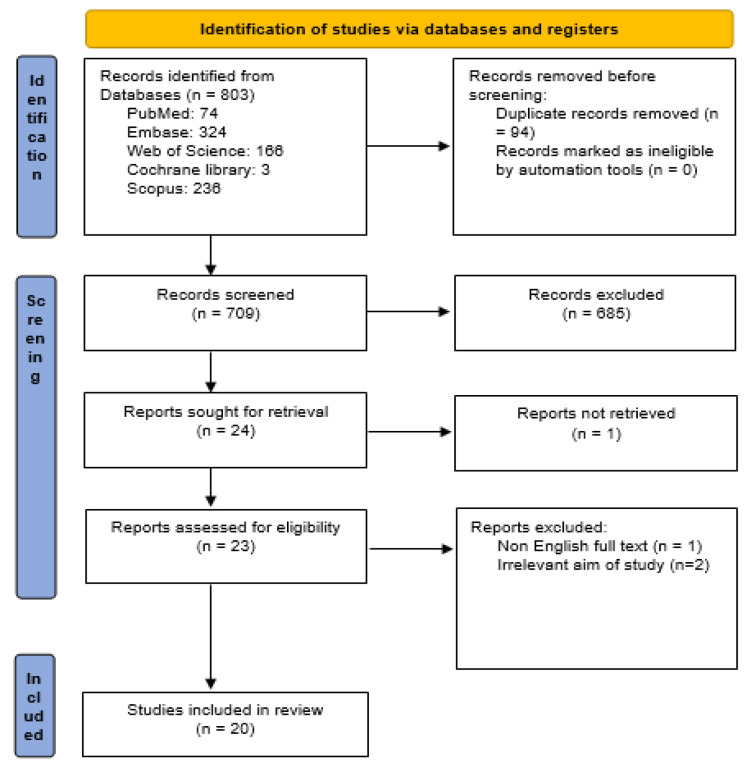


**Table:** 

**Type of Study**	**Models (Patients, animals, cells)**	**Concentration, length of trial**	**Main mechanisms**	**The first author (Reference)**
*In vivo*	Portal vein thrombosis in rats	60 mg/day of RSV was administered per nasogastric tube for ten days	↑ GSH ↓ MDA ┴ Platelet aggregation and coagulation through creases in cAMP levels.	Kirimlioglu (Kirimlioglu et al. 2008)
*In vivo*	Fibrosis of male Sprague-Dawley rats induced by carbon tetrachloride	50 mg/day of RSV was administered per nasogastric tube for ten days	↓ Portal vein system thrombosis, platelet aggregation, and platelet ROS formation ↑ Platelet apoptosis and NO synthesis.	Xu (Xu et al. 2016)
*In vitro*	Human umbilical vein endothelial cells thrombosis-associated markers induced by H_2_O_2_	30 μM of RSV for two hr	↓ P-selectin, PSGL-1, and vWF protein expression by H_2_O_2_ via SIRT1 signaling.	Lou (a) (Lou et al. 2017)
*In vitro*	Human umbilical vein endothelial cells thrombosis-associated markers induced by H_2_O_2_	0, 10, 20 and 30 µM of RSV for 2 hr	↓ ROS levels and suppression of the phosphorylated (p)-p38, P-c-JNKs, and P-ERK via inhibiting MAPK signaling pathways. ↓ Caspase-3, P-selectin glycoprotein ligand-1, and vWF expression.	Lou (b) (Lou et al. 2018b)
*In vivo*, *in vitro* and *ex vivo*	Endothelial progenitor cells and a murine model of venous thrombosis	50 μM of RSV for 48 hr	↑ Thrombus resolution through attenuating miR-138 expression and, therefore, upregulating FAK.	Zhang (Zhang et al. 2018)
*In vivo* and *in vitro*	Rat model of venous thrombosis	25-75 μmol/L of RSV for two days	↑ Progenitor cells angiogenesis through decreased expression of miR-542-3p by targeting ANGPT2, enhanced thrombi recanalization.	Lu (Lu et al. 2019)
*In vivo* and *in vitro*	Male and female Sprague–Dawley rats with stenosis-induced DVT	25, 50, and 75 mg/kg of RSV once daily for two days	↓ The weight of blood clots, the number of white blood cells that entered the area of injury, the levels of two TNF-α and IL-1β, and the levels of two proteins called Ace-p65 and TF. ↑ The levels of SIRT1 and its coding mRNA.	Yao (Yao et al. 2019)
*In vitro*	Thrombosis human umbilical vein endothelial cells	25, 50, and 100 mg/ml of RSV for 24-48 hr	↓ The levels of vWF, t-PA-1, IL-8, the activity of factor VIII, mRNA expression levels of Vwf, t-PA-1, and the intracellular level of t-PA.	Shahidi (Shahidi et al. 2020)
*In vivo*	Thrombosis in DVT SAMP-1 mice model	20 mg/kg of RSV for 14 days	↓ The expression of p16, p21, mRNA, P-selectin, PSGL-1, and Vwf. ↑ P53 and acetylated p53 due to Sirt1 activation.	Lou (c) (Lou et al. 2021)
*In vivo* and *in vitro*	Ferric chloride-induced arterial thrombosis model in rats.	10-50 mg/kg/day of trans-RSV for 8-15 days	, trans-RSV revealed antithrombotic and anti-platelet activities of some antioxidants	Banu (Banu et al. 2022)
*In vivo*	Sprague–Dawley rats with inferior vena cava thrombosis.	15-60 mg/kg for 1 hr	↓ Lesions in the IVC and lung tissue, thrombosis, the levels of D2D, F1 + 2, caspase-1, IL-1β, TF, NLRP3 and HIF-1α	Fei (Fei et al. 2022)

**Table:** 

**Type of Study**	**Models (Patients, animals, cells)**	**Concentration, length of trial**	**Main mechanisms**	**The first author (Reference)**
*In vivo *and* in vitro*	Male Sprague–Dawley adult rats induced PTE by infusion of an autologous blood clot into the pulmonary artery	10 mg/kg/day, i.p of RSV for 1-8 hr	↓ Formation of PTE through down-regulation of the MCP-1 expression by suppressing acute PTE-induced p-p38MAPK activation.	Chun (Park et al. 2012)
*In vivo *and* in vitro*	PE was induced by PAF, AA, ADP, and thrombin in SIRT1-transgenic mice (C57BL/6N) w	Ten μM for one hr of RSV administered to mice (95 μg/kg) or a combination of NAM (5 μg/kg) + RSV (same dose)	↑ SIRT1 ↓ PAFR expression on platelets by proteasomal and lysosomal pathways, ┴ Platelet aggregation in vitro and PE formation *in vivo*.	Kim (Kim et al. 2016)
*In vivo *and* in vitro*	Mouse pulmonary thromboembolism model induced by ADP	150 μM compounds of RSV (2a–f and 4a–f)	┴ Platelet aggregation by releasing NO agonists, ADP, collagen, and arachidonic acid.	Dutra (Dutra et al. 2017)
*In vivo *and* in vitro*	In male Sprague-Dawley adult rats, acute PTE was established in the pulmonary artery.	10 mg/kg/day, i.p. of RSV for 1-8 hr	┴ TNF-α-induced p-p38MAPK expression through down-regulating the MCP-1 expression of RPAs-RSV.	Lin (Lin et al. 2019)
*In vivo, in vitro, *and* insilico*	Male SpragueDawley rats with cardiac injury induced by PE	30 mg/kg of RSV and followed for six weeks	↓ MALAT1/NLRP3 and miR-22-3p expression (following its direct target) ┴ ASC, caspase-1, IL-1β, and IL-18	Yang (Yang et al. 2019)
*In vivo *and* in vitro*	Intravenous injection of ADP in experimental mic and washed human platelets	Pterostilbene at 2 mg/kg was administered	┴ Platelet aggregation, IKK and p65 phosphorylation. ↓ NF-κB signaling pathway, and MK-2206 (an inhibitor of Akt)	Hsia (Hsia et al. 2021)
*In vivo *and* in vitro*	ADP-induced acute pulmonary thromboembolism in male ICR mice using the tail-vein transection model	1−100 μmol/L of pterostilbene in platelets for 20 min and 0.6 and 1.2 mg/kg PTE in mice	↓ P-selectin expression on isolated α-granules inhibited collagen-activated platelets, PLCγ2/ PKC, and MAPK phosphorylation. ┴ ATP production and intracellular ([Ca2+]i) mobilization.	Huang (Huang et al. 2021)
*In vivo* and *in vitro*	Male Sprague Dawley rats with autologous blood clots, pulmonary embolism, and Human Pulmonary Artery Endothelial Cells.	RSV at 10 mg/kg/day concentration for 1, 2, and 4 weeks in rats and 5, 10, or 20 μM in cells	↑ Thrombolysis and pulmonary artery function, reducing mPAP and WA/TA. ┴ IL-6, IL-1β, MPO, TF, vWF, P-selectin, promote SOD in plasma, MCP-1, Ac-FOXO1, VCAM-1, ICAM-1, caspase 3 and 9, and Bax.	Liu (Liu et al. 2022)
RCT	COVID-19 patients	RSV administered two capsules four times per day for at least 7-15 days	Pulmonary embolism incidence rates were equal in both studied groups.	McCreary (McCreary et al. 2022)

## Discussion

This systematic review investigated the effects of RSV on VTE, focusing on the mechanisms as follows:

### Antioxidant effects

Oxidative stress is a complex phenomenon when the generation of reactive oxygen species (ROS) surpasses the body's ability to detoxify or repair the damage (Pizzino et al. 2017). Studies have demonstrated that polyphenols provide beneficial effects by decreasing endothelial dysfunction, platelet activation, oxidative stress in the vascular wall, overactivity of the local angiotensin system, and heightened prothrombotic reactions due to cardiovascular diseases (Oak et al. 2018; Tromba et al. 2019). It has been proposed that oxidative stress may contribute to thrombotic events through several mechanisms. 

Oxidative stress and inflammation significantly impact the endothelial cells, shifting their function from a vasoprotective, prothrombotic, and pro-apoptotic state to a vasoconstrictive one (Drożdż et al. 2023). ROS significantly reduces nitric oxide (NO) production while oxidative stress, inflammation, and endothelial dysfunction exacerbate the damaging process (Gaur et al. 2024). In this regard, an *in vivo* study, conducted by Xu et al., revealed that 50 mg/day of RSV administration through nasogastric tube for ten days in fibrosis of male Sprague-Dawley rats induced by carbon tetrachloride could reduce portal vein system thrombosis, platelet ROS formation, and improved platelet NO synthesis (Xu et al. 2016). Another study by Lou et al. reported that applying 30 µM of RSV for two hr on human umbilical vein endothelial cells thrombosis-associated markers induced by H2O2 could downregulate thrombosis-associated markers through reduced ROS levels (Lou et al. 2018b)

Prooxidant stimulants such as ROS, angiotensin II, Endothelin-1 (ET-1), and inflammatory cells tend to dominate the antioxidant defense and can lead to oxidative stress (Cushnie et al. 2024). This stress triggers inflammation, which results in endothelial dysfunction and vascular tone regulation impairment. Furthermore, it increases the susceptibility to forming foam cells and adverse vascular remodeling (D'Oria et al. 2020; Guo et al. 2014; Moris et al. 2017).

Moreover, oxidative stress has been linked to increased platelet activation and aggregation, which are critical players in the formation of blood clots. Elevated ROS levels may enhance platelet reactivity, promoting thrombus formation. Oxidative stress may also influence the activation of various coagulation cascade components, including clotting factors and fibrinolysis, and involve various cell types and anticoagulant pathways (Bettiol et al. 2022; Wang and Zennadi 2020). The intricate interplay between ROS and mitochondria is the critical regulator of platelet functions. A profound shift in platelets' redox balance and metabolism occurs upon activation. Upon activation, multiple signaling pathways stimulate the production of ROS by NADPH oxidase (NOX) and mitochondria. These ROS molecules derived from platelets act as a catalyst, further amplifying the production of ROS and triggering a self-propagating loop of platelet activation, adhesion, and recruitment (Masselli et al. 2020).

Furthermore, the accumulation of ROS within red blood cells (RBCs) is caused by the constant autoxidation of endogenous hemoglobin (Hb) and trigger of NADPH oxidase, as well as the absorption of extracellular ROS formed by other cells in circulation. This accumulation can increase RBC ROS levels, ultimately affecting the structure and function of the RBC membrane (Wang and Zennadi 2020). Abnormal erythrocytes, also known as RBC, which are present in certain medical conditions, tend to attach to the inner lining of blood vessels. This attachment can contribute to the generation of thrombin, a protein involved in blood clotting, and the formation of a thrombus, or blood clot. Such a process can lead to an accelerated breakdown of RBC, a condition known as hemolysis, and a hypercoagulable state where the blood has an increased tendency to clot (Bettiol et al. 2022).

Antioxidant enzymes play a critical role in protecting our bodies from the harmful effects of unstable molecules, also known as free radicals. These enzymes compromise glutathione peroxidase (GPx), superoxide dismutase (SOD), and catalase (CAT). SOD and CAT, in particular, act as the primary defense system against free radicals by neutralizing two of the most common types of ROS, including O2- and hydrogen peroxide (H_2_O_2_). This process of scavenging ROS is vital for maintaining the delicate balance of our cells and preventing oxidative stress (Pham-Huy et al. 2008). On the other hand, when a tissue is subjected to oxidative stress due to the presence of H_2_O_2_, it can cause changes in the activities of CAT, GPx, NO synthase, and its levels. However, it has been found that RSV treatment can help prevent these changes (Konyalioglu et al. 2013). Kirimlioglu et al. (Kirimlioglu et al. 2008) and Liu et al. (Liu et al. 2022) in their studies indicated that 60 mg/day of RSV was administered per nasogastric tube for ten days and 10 mg/kg/day of RSV concentration for 1, 2, and 4 weeks in rats, respectively, can increase GSH and decrease MDA and SOD in plasma.

Moreover, RSV is considered a Sirtuin 1 (SIRT1) activator. The primary responsibility of SIRT1 is to respond to oxidative stress by regulating several transcription factors, including forkhead box protein O1 (FOXO1), FOXO3a, FOXO4, peroxisome proliferator-activated receptor gamma coactivator 1-alpha (PGC-1α) and nuclear factor kappa B (NF-κB) regulators. These transcription factors work in concert to activate the expression of antioxidant enzymes and eliminate ROS (Gu et al. 2021).

A study indicated that 30 μM of RSV has been found to have a promising effect on venous thrombosis. It can reduce the activation of thrombosis-related markers caused by H_2_O_2_ by activating SIRT1, a protein that plays a crucial role in cellular metabolism and stress response (Lou et al. 2017)

Lastly, oxidative stress might influence the composition and stability of blood clots, given the changes in the redox state within the thrombus, which may affect its susceptibility to lysis and resolution (Izzo et al. 2021b).

Oxidative stress, which leads to increased levels of ROS, is vital in altering the extracellular matrix (ECM) turnover and metabolism. The ECM is responsible for providing structural support to the cells and tissues. Any disturbance in its balance can lead to various pathological conditions. In human aging hearts with atrial fibrillation, matrix metalloproteinase (MMP) levels, a family of enzymes involved in ECM degradation, are significantly elevated (Zhan et al. 2018). 

### Anti-inflammatory effects

Inflammation plays a complex role in the pathophysiology of DVT and PE, involving multiple mechanisms. The close relationship between inflammation and thrombosis is essential for containing the spread of pathogens through immunothrombosis. However, excessive activation or dysregulation of this mechanism can lead to thromboinflammation, which causes tissue ischemia due to thrombi formation (Marcos-Jubilar et al. 2023).

The main factor behind these processes is the vicious cycle of platelet and innate immune cell activation, the complement system, and the coagulation cascade. Clinical evidence supports the connection between inflammation and thrombosis, as various conditions such as chronic autoimmune diseases, infections, and hematopoiesis of indeterminate potential are associated with a higher risk of thrombotic events (Stark and Massberg 2021).

Inflammation and oxidative stress are also interconnected, where oxidative stress can trigger inflammatory responses, and inflammation, in turn, can contribute to oxidative stress. Chronic inflammation, a risk factor for DVT and PE, may be influenced by oxidative stress (Siti et al. 2015). 

Numerous intracellular pathways are known to be activated in endothelial cells, leading to phenotypical changes that impart an inflammatory cell-like appearance.

Recent evidence suggests that endothelial-to-mesenchymal transition (EndMT) plays a crucial role in endothelial dysfunction associated with inflammation and thrombosis (Pilard et al. 2022). In response to growth factors and cytokines such as thrombin, tumor necrosis factor-alpha (TNF-α), interleukin-1 (IL-1), and insulin, an increase in ET-1 synthesis is also observed.

Similarly, vasoactive substances, including norepinephrine, angiotensin II, vasopressin, and bradykinin, also increase ET-1 synthesis (Genovesi et al. 2022). Elevated ET-1 and platelet-activating factor (PAF) synthesis leads to vasoconstriction and enhances the prothrombotic state (Gross and Aird 2000). Additionally, Increased levels of C-reactive protein (CRP), IL-6, IL-8, and TNF-α during systemic inflammation have been associated with a higher risk of VTE (Branchford and Carpenter 2018; Poredos and Jezovnik 2007). * In vitro* and *in vivo* studies investigating RSV effects on proinflammatory cytokines have shown that RSV can reduce IL-8 IL-,1β, and IL18 in DVT and PE situations (Shahidi et al. 2020; Yang et al. 2019). An *in vivo* and *in vitro *study by Yao et al. demonstrated that administration of 25 mg/kg of RSV once daily for two days in male and female Sprague–Dawley rats with stenosis-induced DVT can decrease the number of white blood cells that entered the area of injury, and lower the levels of two TNF-α and IL-1β (Yao et al. 2019). Another study indicated that administration of 10 mg/kg/day of RSV for 1-8 hr in male Sprague-Dawley adult rats with acute PE could inhibit TNF-α-induced p-p38MAPK expression through downregulation of monocyte chemoattractant protein-1 (MCP-1) (Lin et al. 2019). The NF-κB pathway, which regulates proinflammatory cytokine production, was inhibited by pterostilbene (natural polyphenol and a stilbenoid chemically related to RSV) at 2 mg/kg administration in rats (Hsia et al. 2021).

The nucleotide-binding domain leucine-rich repeat-containing protein 3 (NLRP3) inflammasome plays a fundamental role in producing and maturing IL-1β, a proinflammatory cytokine. It requires the integration of two distinct signals. The initial priming signal triggers the transcription and translation of NLRP3 and pro-IL-1β via a signaling cascade involving the Toll-like receptor-nuclear factor-kappa B (TLR-NF-κB) pathway (Lebreton et al. 2018). Fei et al. reported that 60 mg/kg of RSV for 1 hr in Sprague–Dawley rats with inferior vena cava thrombosis could reduce the expression of NLRP3 and hypoxia-inducible factor-1α (HIF-1α) (Fei et al. 2022). Another study indicated that 30 mg/kg of RSV, after six weeks, reduced MALAT1/NLRP3 and miR-22-3p expressions (Yang et al. 2019). 

Monocyte chemoattractant protein-1 (MCP-1) is also a potent inflammatory cytokine associated with thrombosis formation (Chen et al. 2020). Studies suggest that measuring plasma levels of MCP-1 can provide valuable prognostic information in cardiac diseases and might be considered independent of standard clinical variables. MCP-1 may have prothrombotic effects, increasing the risk of DVT and PE (Gonzalez-Quesada and Frangogiannis 2009; Humphries et al. 1999). Two *in vivo* and *in vitro *studies by Chun et al. and Lin et al. indicated that 10 mg/kg/day, intraperitoneal (i.p) of RSV for 1-8 hr in male Sprague-Dawley adult rats acute PE inhibited TNF-α-induced p-p38MAPK expression through downregulating MCP-1 expression of rat pulmonary artery endothelial cells (RPAs)-RSV (Lin et al. 2019; Park et al. 2012).

The coagulation system can be activated by the induction of tissue factor (TF) by activating endothelial cells, platelets, and leukocytes, which then form microparticles (Branchford and Carpenter 2018). The activation of TF has been demonstrated to elicit a clotting response in venous thrombosis. Furthermore, the presence of inflammation exacerbates TF activity in triggering exogenous coagulation pathways, thereby resulting in the formation of extensive fibrin in venous vessels (Müller et al. 2003). Liu et al. reported that RSV at 10 mg/kg/day promoted thrombolysis and pulmonary artery function by inhibiting TF in male Sprague Dawley rats with autologous blood clots and pulmonary embolism (Liu et al. 2022). RSV at 60 mg/kg in Sprague–Dawley rats with inferior vena cava thrombosis could attenuate lesions in the inferior vena cava lung tissue and the thrombosis was attenuated in the RSV group by reducing TF (Fei et al. 2022). Yao et al. revealed that the administration of 25 mg/kg of RSV once daily for two days in male and female Sprague-Dawley rats with stenosis-induced DVT decreased the number of white blood cells that entered the area of injury and lowered the levels of two proteins called Ace-p65 and TF (Yao et al. 2019).

In addition, inflammation can recruit neutrophils and lead to the formation of neutrophil extracellular traps (NETs), contributing to thrombus formation (Zhou et al. 2021). Neutrophils and NETs play a critical role in the formation of thrombosis. Upon being stimulated by pathogens, neutrophils release extracellular structures known as NETs. These structures contain active substances such as neutrophil elastase (NE), myeloperoxidase (MPO), and cathepsin G. NETs form a network structure that can prevent the spread of pathogens, effectively killing and eliminating them. However, the components of NETs can also activate the coagulation pathway abnormally, contributing to the formation of pathological thrombus (Zhou et al. 2022). NETs are complex structures that play a crucial role in developing DVT. These specialized formations are released by neutrophils, which can effectively capture harmful pathogens and facilitate clotting within the body's deep veins (Yao et al. 2023).

According to recent studies, an imbalance in the vascular microenvironment and the overproduction of NETs can result in abnormal thrombosis. NETs serve as a structure for platelets, RBCs, and coagulant molecules, which can lead to thrombosis. Additionally, the protein constituents found in NETs can activate the body's natural coagulation process, ultimately leading to thrombosis. Therefore, NETs are essential in developing arterial, venous, and cancer-related thrombosis (Zhou et al. 2021). In thrombosis formation, the von Willebrand Factor (vWF) plays a crucial role in preserving DNA and elevating serum DNA levels.

Consequently, specific markers of NETs, such as extracellular DNA and citrullinated histone H3 (CitH3), may be present within the thrombus (El-Sayed et al. 2016). Research has shown that Monocyte Extracellular Traps (ETs) have a similar structure to NETs and contain substances such as MPO, lactoferrin, citrullinated histones, and elastase, which can increase the risk of clotting (Granger et al. 2017). Exosomal MPO, considered a biomarker of DVT, increased sharply in animals with inferior vena cava ligation (Han et al. 2022). Another study indicated that RSV promoted thrombolysis and pulmonary artery function by inhibiting MPO in male Sprague Dawley rats with autologous blood clots and pulmonary embolism (Liu et al. 2022). 

Caspase-1 is an enzyme present in different cell types, including immune and non-immune cells. When activated, it triggers the formation of gasdermin D, which acts as a protein channel or pore that allows the release of cytokines IL-1β and IL-18. These cytokines are essential in the body's immune response and inflammatory processes (Yang et al. 2019). Caspase-1 is thought to primarily regulate cell death and protein secretion while potentially affecting lysosomal function that varies depending on the specific cell type (Molla et al. 2020). Administration of 30 mg/kg of RSV for six weeks resulted in a significant reduction in the expression levels of caspase-1, IL-1β, and IL-18, indicating its potential anti-inflammatory effects (Yang et al. 2019).

Inflammatory mediators can also downregulate natural anticoagulant pathways, exacerbating the risk of thrombotic events. When exposed to endotoxin and inflammatory agents, endothelial cells are prompted to express TF and factor XII. This leads to the activation of the coagulation pathway and consumption of natural coagulation inhibitors like antithrombin III, protein C, and protein S (Landau et al. 2022; van Hinsbergh 2012).

### Anti-apoptosis effects

Thrombi form when specific coagulation factors, such as fibrinogen and factor XIIa, are triggered on biomaterials in conjunction with TF from inflammatory cells like neutrophils and monocytes. The process of platelet activation leads to the conversion of native CRP into monomeric C-reactive protein (mCRP). In addition, microparticles generated by apoptotic and activated cells and platelets can separate native CRP into mCRP and contain TF. When cells are present in the bloodstream, they attach themselves to protein surfaces and enter the biomaterial, producing MMPs and ROS and triggering inflammation (Labarrere et al. 2020). So, proinflammatory stimuli, such as TNF-α, CRP, TF, and interferon-gamma (IFN-γ), may compromise cell survival pathways in monocytes, endothelial cells, and platelets (Affara et al. 2007; Mallat and Tedgui 2000; Nan et al. 2023). Therefore, these inflammatory and oxidative pathways precipitate apoptotic trends during VTE.

Moreover, the balance of endothelial cell growth and decay is essential for creating new vessels and the regression of preexisting ones during both normal development and disease (Mallat and Tedgui 2000). The connection between cells via the VE-cadherin pathway is crucial for the survival of growth factors such as vascular endothelial growth factor (VEGF). Shear stress can increase the level of VE-cadherin expression. The process of Akt phosphorylation plays a crucial role in several signaling pathways associated with anti-apoptotic effects, which in turn affect the equilibrium between pro-apoptotic and anti-apoptotic members of the bcl-2 family (Mallat and Tedgui 2000; Nan et al. 2023).

Lou et al. demonstrated that treatment with 30 µM of RSV on human umbilical vein endothelial cells, which were exposed to H2O2 for 2 hours and associated with thrombosis markers, inhibited the phosphorylation of p38, c-Jun N-terminal kinases (c-Jun) and extracellular signal-regulated kinases (ERK). This effect was attributed to the inhibition of the MAPK signaling pathways (Lou et al. 2018b). P53 can induce apoptosis in cells commonly by direct transcriptional activation of the pro-apoptotic pathways (Lebreton et al. 2018). In Lou et al. study (Lou et al. 2021), RSV reduced the expression of p16 and p21 and increased p53 and acetylated p53 due to SIRT1 activation by administration of 20 mg/kg of RSV for 14 days in thrombosis in the DVT SAMP-1 mice model. Moreover, the application of RSV in male Sprague Dawley rats with autologous blood clot-induced PE inhibited caspase-3, caspase-9, and Bcl-2-associated X protein (BAX) (Liu et al. 2022).

### Anticoagulation and anti-platelet activity

The coagulation system is critical in developing VTE. 

The initiation of the coagulation cascade is a complex process that involves several pathways and mechanisms. Typically, coagulation is initiated in response to vascular injury or activation. When blood vessels are damaged, tissues release TF, a vital initiator of the extrinsic coagulation pathway. The extrinsic pathway is triggered by TF, forming a complex with factor VII which activates factors IX and X, leading to the conversion of prothrombin to thrombin. Simultaneously, the intrinsic pathway is activated within the bloodstream, involving factors XII, XI, IX, and VIII (Palta et al. 2014). As age develops, there is typically a gradual increase in the concentration of specific coagulation factors found in plasma, including factor V, VII, VIII, IX, and fibrinogen. A similar trend is observed with vWF, a vital protein responsible for the interaction between platelets and the walls of blood vessels. The elderly population has a greater risk of cardiovascular events, which could be related to higher levels of plasma fibrinogen (Franchini 2006). This protein aids in platelet bridging through the glycoprotein IIb-IIIa receptor and is a direct substrate for clotting. Moreover, the elevated levels of fibrinogen can lead to an increase in blood viscosity and thrombosis (Previtali et al. 2011). Shahidi et al. indicated that 25, 50, and 100 mg/ml of RSV for 24-48 hr on thrombosis in human umbilical vein endothelial cells attenuated the levels of vWF and t-PA-1as well as the activity of factor VIII (Shahidi et al. 2020).

The coagulation cascade involves thrombin as a key enzyme which facilitates the conversion of fibrinogen to fibrin. Fibrin forms a mesh that helps in stabilizing the blood clot. Platelets play a crucial role in developing VTE, as they are activated and aggregated together to form a plug at the site of vascular injury. Activated platelets also provide a surface for the coagulation cascade, facilitating the conversion of prothrombin to thrombin (Vrotniakaite-Bajerciene et al. 2023). Fibrin strands generated by thrombin weave through the platelet plug, forming a stable blood clot. This process, known as secondary hemostasis, reinforces the initial platelet plug and prevents bleeding. While the coagulation system promotes clot formation, anticoagulant mechanisms act to prevent excessive coagulation. Fibrinolysis breaks down fibrin clots, which helps prevent clot propagation and promotes clot resolution (Periayah et al. 2017). Certain genetic and acquired conditions can lead to a hypercoagulable state, making individuals more prone to thrombosis. Venous stasis, or slow blood flow, is a risk factor for DVT, and if a clot in the deep veins dislodges and travels to the lungs, it can cause a PE, obstructing blood flow. Moreover, D-dimer (D2D) and prothrombin fragment 1+2 (F1+2) levels were reduced following the administration of 15–60 mg/kg of RSV treatment for one hour in Sprague–Dawley rats with inferior vena cava thrombosis (Fei et al. 2022).

Platelets play a crucial role in the interplay between thrombosis and inflammation, two essential aspects of hemolysis-associated disorders. Platelets require pattern recognition receptors, such as toll-like receptor 4 (TLR4) and NLRP3 (Vogel and Thein 2018), to fulfill this role. NLRP3 forms multiprotein inflammasome complexes within platelets.

Additionally, platelets are susceptible to damage-associated molecular pattern (DAMP) molecules such as free haem and high-mobility group box 1 (HMGB1), which can target and affect them. HMGB1 is a DNA-binding protein, released by activated platelets and dying or stressed cells, while free haem is a by-product occurring during hemolysis from Hb oxidation. Platelet TLR4, NLRP3, and Bruton tyrosine kinase (BTK) play a crucial role in regulating platelet aggregation and thrombus formation (Vogel and Thein 2018). 

### Adhesion molecules

Another mechanism contributing to VTE is leukocyte adhesion and infiltration, which is facilitated by the expression of adhesion molecules on endothelial cells. Leukocytes can release proinflammatory factors, contributing to forming a prothrombotic environment (Yao et al. 2023). 

Specific proteins, including vascular cell adhesion molecule-1 (VCAM-1), P-selectin, complement components, and fibrinogen, can adhere to biomaterials circulating in the body. This protein adhesion promotes the attachment of neutrophils and macrophages to the biomaterial. Macrophages use various integrins on their surfaces to attach to protein-coated biomaterials like vitronectin, fibronectin, Factor Xa, fibrinogen, complement components, and other inflammatory cell surfaces (Labarrere et al. 2020). Liu et al. in their study showed that RSV administration (10 mg/kg/day concentration) in rats inhibited MCP-1, Ac-FOXO1, VCAM-1, and intercellular adhesion molecule 1 (ICAM-1) (Liu et al. 2022). 

Neutrophils are the most prevalent leukocyte found in venous thrombosis. While they can contribute to damage in the vein walls, they also serve a vital role in regulating the production and activity of fibrinolytic enzymes, promoting the breakdown of fibrin and collagen, and are essential for early thrombus lysis (Longstaff and Kolev 2015). Plasmin is a crucial component of thrombolysis, particularly in the early stages of venous thrombolysis. Meanwhile, D-dimer is a degradation product of fibrinolysis that is clinically significant due to its ability to diagnose, assess efficacy, and predict prognosis in cases of thrombotic disease. Additionally, neutrophils can secrete MMP, activating the plasminogen activation system and reducing the accumulation of inflammatory factors in DVT formation (Yao et al. 2023).

Moreover, following blood vessel injury, platelets adhere to exposed collagen at the injury site and become activated, releasing adenosine diphosphate (ADP) and thromboxane A₂ (TXA₂). These molecules act as potent platelet agonists, amplifying platelet recruitment and aggregation to form a hemostatic plug, effectively inhibiting the bleeding (Caillon et al. 2022). Platelets and RBCs are the primary components of a thrombus, which can form in a flowing or static blood environment. After a few days, inflammatory cells such as neutrophils, lymphocytes, and monocytes infiltrate the margins of the thrombus, leading to an inflammatory response. This process depends on endothelial activation and can increase the expression of cell adhesion molecules, including p-selectin, e-selectin, and vWF (Ley et al. 2007).

The P-selectin/PSGL-1 pathway is also responsible for critical events in starting and spreading venous thrombosis. It makes it easier for leukocytes and platelets to accumulate within the growing thrombus. Both activated platelets and endothelium contain P-selectin and bind to P-selectin glycoprotein ligand-1 (PSGL-1) in all leukocytes (Wong et al. 2021). A highly potent P-selectin inhibitor called PEG40-GSnP-6 (P-G6) has been created to counter this. P-G6 has been shown to inhibit platelet-monocyte and platelet-neutrophil aggregation *in vitro* and blocks microcirculatory platelet-leukocyte interactions *in vivo* (Wong et al. 2021). Studies have shown that 10, 20, and 30 μM of RSV for 2 hr attenuated P-selectin and glycoprotein ligand-1, PSGL-1, and vWF protein expression (Liu et al. 2022; Lou et al. 2017; Lou et al. 2018b; Lou et al. 2021; Shahidi et al. 2020).

### Vasodilation and endothelial protection

Various factors have important roles in PE and DVT incidence, including activated platelets, endothelial effects, reflexes, and hypoxia. This interaction leads to vasoconstriction (Lyhne et al. 2020). NO primarily induces vasodilation through the endothelial NO synthase (eNOS)/NO/cyclic guanosine monophosphate (cGMP) pathway activity. This pathway is the primary mechanism through which, NO exerts its vasodilatory effects. In the glomeruli, eNOS is predominantly expressed in the endothelial cells, crucial in regulating blood flow (Ahmad et al. 2018). When NO binds to soluble guanylate cyclase (sGC), it activates the enzyme and triggers the conversion of guanosine triphosphate (GTP) to cyclic guanosine monophosphate (cGMP). 

In two *in vivo* and *in vitro* studies, RSV improved platelet apoptosis and NO synthesis and released NO agonists ADP (Dutra et al. 2017; Xu et al. 2016).

RSV has potential cardiovascular benefits, including vasodilation and endothelial protection, that may reduce the risk of VTE (Gal et al. 2021). RSV enhances NO synthesis in endothelial cells, a potent vasodilator that relaxes vascular smooth muscle cells, leading to increased blood flow and improved endothelial function. It activates eNOS, the enzyme responsible for NO production in endothelial cells, and supports NO generation, contributing to vasodilation (Li et al. 2019). RSV-induced NO production may enhance the cGMP pathway by activating SIRT1 which leads to the relaxation of vascular smooth muscle, contributing to vasodilation (Fukuhara et al. 2011). RSV has been shown to decrease the production of ET-1, a vasoconstrictor, which may contribute to vasodilation and prevent excessive vasoconstriction. RSV may improve overall endothelial function by supporting the production of vasodilators, reducing vasoconstrictors, and enhancing antioxidative defense mechanisms (Gordish and Beierwaltes 2014; Li et al. 2019).

RSV modulates calcium channels by directly inhibiting the L-type voltage-gated calcium channels (VGCC), which influences intracellular calcium levels, affecting smooth muscle contractility and promoting vasodilation (McCalley et al. 2014). It also activates potassium channels, leading to cell membrane hyperpolarization, reducing smooth muscle cell excitability, and contributing to vasodilation. RSV primarily targets the plasma membrane. It also indirectly affects the large conductance calcium-activated potassium (BK) channel, improving the endothelium and vascular function (Protić et al. 2013).

Furthermore, angiopoietin-2 (ANGPT2) is a protein that plays a crucial role in regulating the formation of new blood vessels (angiogenesis) and the resolution of blood clots in veins. However, when ANGPT2 is overproduced, it can lead to the development of thrombo-fibrosis, a condition characterized by the formation of fibrous tissue within blood clots (Hobohm et al. 2021). EPCs were treated with 25 μmol/L of RSV for 2 days. The presence of miR-542-3p led to a reduction in luciferase activity. Furthermore, results from western blot and RT-PCR analyses demonstrated that miR-542-3p affected the expression of ANGPT2 at the post-transcriptional level (Lu et al. 2019) .

### Activating SIRT1

SIRT1, belonging to the sirtuin family of NAD+-dependent deacetylases, has been found to play a crucial role in various cellular processes such as metabolism, stress response, and aging (Bettiol et al. 2023). Studies have shown that Sirt1, a protein that regulates gene expression, is connected to various medical conditions, including inflammation, oxidative stress, platelet adhesion, and endothelial cell senescence (Huang et al. 2023; Lou et al. 2021; Pan et al. 2022). SIRT1-AS, a type of RNA that works in opposition to SIRT1, is a crucial factor in developing DVT due to its association with decreased SIRT1-AS and SIRT1 lncRNA expression. By slowing down aging and reducing the likelihood of blood clot formation, SIRT1 plays a vital role in preventing DVT. Additionally, the SIRT1-AS antisense RNA helps to reduce the risk of DVT by regulating the SIRT1/FOXO3a pathway (Huang et al. 2023; Lou et al. 2021). SIRT1 is crucial in regulating thrombosis and modulating key pathways like endothelial activation, coagulation, and platelet aggregation.

Moreover, SIRT1 inserts anti-inflammatory properties primarily by reducing oxidative stress (Bettiol et al. 2023). Studies have suggested several hypothetical mechanisms through which RSV may influence SIRT1 activation and impact the incidence of DVT and PE (Lou et al. 2017; Lou et al. 2021; Yao et al. 2019). A study observed that a higher degree of DVT is linked to more significant endothelium senescence and lower anti-aging gene SIRT expression. *In vivo* experiments revealed that overexpression of SIRT1 can prevent endothelial senescence and reduce the incidence of DVT. Also, the study found that its expression is low in the endothelium of severe thrombosis. Overexpressing lncRNA SIRT1-AS upregulated SIRT1, reduced the expression of senescence and DVT-related biomarkers in human vascular endothelial cells (HUVECs) (Lou et al. 2019). Recent studies suggest that the endothelial SIRT1 enzyme is vital in mitigating the harmful effects of hypoxia-induced endothelial dysfunction and thrombotic inflammation in DVT. This beneficial effect is achieved through the deacetylation of NF-κB, a protein that plays a significant role in inflammatory processes. Essentially, the presence of endothelial SIRT1 helps to reduce the activity of NF-κB, which in turn helps to alleviate the negative impacts of hypoxia-induced endothelial dysfunction and thrombotic inflammation in DVT (Tang et al. 2023).

RSV is also effective in mitigating intestinal ischemia-reperfusion injury by increasing SIRT3 expression and decreasing ferroptosis. Via activating the SIRT3/FoxO3a pathway, RSV enhances the SOD and CAT levels, minimizes ROS and LPO production, compensates for the GSH/GPX4 pathway, and inhibits ferroptosis. This promising research suggests that RSV has the potential to be a valuable tool in reducing intestinal ischemia-reperfusion injury (Wang et al. 2023). After being exposed to thrombin, the levels of MCP-1, VCAM-1, ICAM-1, cleaved caspase 3, cleaved caspase 9, and Bax protein are increased (Liu et al. 2022). On the contrary, RSV caused a rise in the quantities of SIRT1 protein, which is recognized for its ability to protect against inflammation. Moreover, RSV caused an increase in the levels of messenger ribonucleic acid (mRNA) that encodes SIRT1 (Yao et al. 2019). The regulation of p53 activity is of great interest in the context of DVT, a potentially life-threatening condition characterized by the formation of blood clots in the deep veins of the body.

Intriguingly, recent studies have demonstrated that acetylated p53 levels are elevated in DVT models, suggesting a potential role for this modification in the pathogenesis of the disease. However, treatment with RSV, a natural polyphenolic compound known to activate SIRT1, significantly decreases acetylated p53 levels in DVT models. These findings suggest that SIRT1-mediated deacetylation of p53 may represent a potential therapeutic target for DVT treatment (Lou et al. 2021). One key factor that regulates platelet activity is cyclic AMP (cAMP). This molecule plays an important role in preventing platelet aggregation. A recent study by Park and colleagues found that RSV can directly inhibit cAMP-dependent phosphodiesterases. This inhibition sets off a series of events that ultimately affect two important metabolic regulators, AMPK and SIRT1 (Park et al. 2012). Various studies indicated that RSV attenuated P-selectin, PSGL-1, and vWF protein expression by H_2_O_2_ increase p53 and acetylated p53 and PAFR expression on platelets via SIRT1 signaling (Kim et al. 2016; Lou et al. 2017; Lou et al. 2021).

In general, the efficacy and mechanisms of RSV in the treatment of VTE are summarized in Figure 2.

**Figure:**
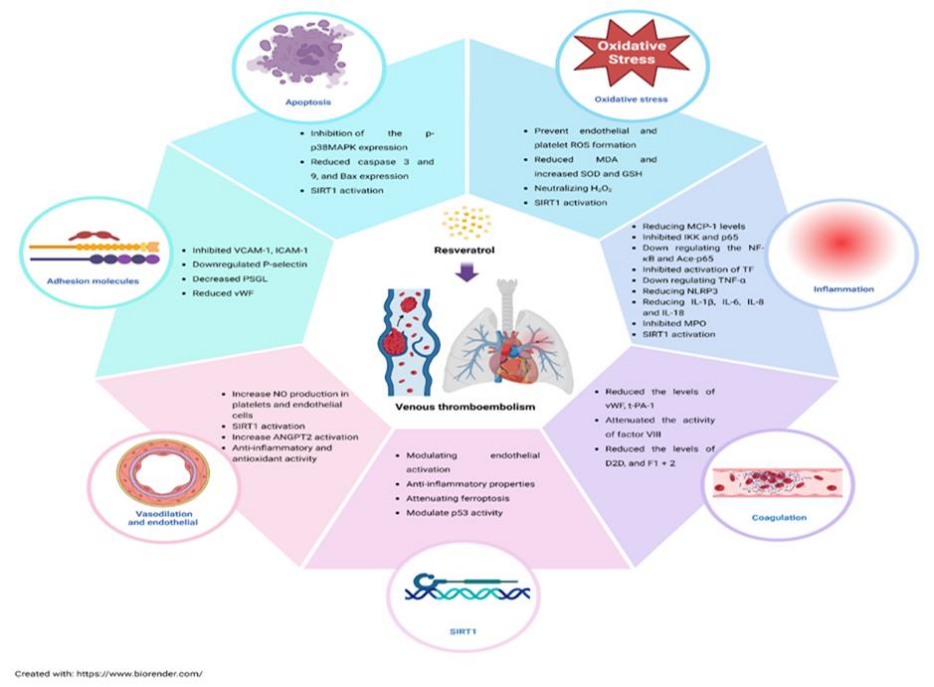


Most studies have preclinical designs and involve animal models or *in vitro* experiments. More well-designed controlled clinical trials are needed, which is one of the limitations of translating the findings to clinical practice. For the clinical use of RSV in the treatment of VTE, its long-term use and different doses should be investigated, and its drug interactions with other drugs and its side effects should be investigated more deeply.

Moreover, the included studies vary significantly in design, methodology, and patient populations. Heterogeneity across studies can make it challenging to draw consistent conclusions and may limit the generalizability of findings. There needs to be standardized dosages and treatment durations across studies. Variability in the administered doses of RSV and the duration of interventions makes it challenging to establish optimal therapeutic regimens.

*In vivo* and *in vitro* studies revealed that RSV has promising effects on VTE through antioxidant, anti-inflammatory, anti-apoptosis, anticoagulation, and antiplatelet activity and inhibits adhesion molecules and vasodilation and endothelial protection properties. RSV has significant potential as a therapeutic agent for VTE prevention and treatment. However, further clinical investigations are necessary to confirm its effectiveness, establish standardized dosages, and ensure its safe integration into clinical practice. 
